# Case report: A case of acute renal failure, rhabdomyolysis, and toxic encephalopathy associated with diquat poisoning in a pregnant woman

**DOI:** 10.3389/fmed.2025.1533841

**Published:** 2025-02-07

**Authors:** Mengqin Li, Bao Qin, Yuan Chen, Yan Cui, Ying Chun Hu, Zhi Jiang

**Affiliations:** ^1^Affiliated Hospital of North Sichuan Medical College, Nanchong, China; ^2^Dazhou Central Hospital, Dazhou, Sichuan, China; ^3^Hospital of Southwest Medical University, Luzhou, China

**Keywords:** diquat, diquat poison, rhabdomyolysis, toxic encephalopathy, abortion, acute kidney injury

## Abstract

We studied the case of a 22-year-old Pregnant woman who self-administered about 200 ml of diquat solution (200 g/L) during a suicide attempt. She developed vomiting, and was admitted to the emergency department of our hospital 5 h later. Based on symptoms, and elevated levels of myoglobin and creatine kinase, the patient was diagnosed with rhabdomyolysis, acute renal failure and toxic encephalopathy caused by diquat poisoning. Undergoing hemoperfusion and hemofiltration were administered immediately. Rhabdomyolysis, toxic encephalopathy, and abortion occurred successively in the pregnant woman. The patient was discharged from the hospital after 37 days of treatment with a Glasgow–Pittsburgh Cerebral Classification (CPC) score of grade 2.this was a case of diquat poisoning complicated with renal failure, rhabdomyolysis, and toxic encephalopathy in a pregnant, which would enrich the experience of diquat poisoning treatment.

## 1 Introduction

Diquat is a bipyridine-inactivating herbicide with strong toxic effects (1). It can be absorbed through the digestive tract, respiratory tract, eyes, or cutaneous mucosa, and is excreted mainly by the kidneys. Patients may experience oliguria, anuria, and other AKI manifestations during the early stages of poisoning. Multiple organ damage may also occur with disease progression, leading to fatal outcomes (2). At present, no specific antidote exists for diquat poisoning, and the main treatment methods include removing the unabsorbed poison and accelerating its excretion of the absorbed poison. Rhabdomyolysis, toxic encephalopathy, and abortion occurred successively in the pregnant woman. This article presents the case of a pregnant woman who was treated for diquat poisoning.

## 2 Clinical information

A 22-year-old woman was admitted to the emergency department due to intentional ingestion of diquat in September 2023 for “self-administration of diquat for over 5 h.” The patient had orally ingested approximately 200 mL of Diquat five or more hours before admission. Subsequently, the patient developed nausea and vomiting. She was sent to a local hospital for gastric lavage1 h prior and was then transferred to our department. Multiple suicide attempts had been made previously. The patient was 20 weeks pregnant at the time of admission. Physical examination revealed the following findings: T: 36.6°C, P: 92 bpm, R: 16 bpm, BP: 100/63 mmHg. The patient was conscious, and no abnormal signs were observed during the examination of the heart, lungs, and abdomen. Bilateral lower limbs showed no edema. At the time of admission, laboratory examinations showed the following: blood gas analysis: pH 7.40, partial pressure of oxygen (PaO_2_) 115 mmHg, partial pressure of carbon dioxide (PaCO_2_) 27 mmHg, oxygen saturation 99.2%; blood routine: leukocytes 20.17 × 10^9^/L, neutrophil percentage 95.8%, erythrocytes 3.89 × 10^12^/L, hemoglobin 122 g/L, platelets 177 × 10/L, liver and kidney function: ALT 20 U/L, AST 58 U/L, total bilirubin 8.5 μmol/L, creatinine 66.6 μmol/L. She was diagnosed with diquat poisoning based on her medical history after admission, and activated charcoal with mannitol was administered orally. Anti-inflammatory, acid-suppressing, and antioxidant treatments were administered immediately.

The patient developed thoracodynia on the day of admission. Examination of abdominal distension: The uterine floor was located at the level of the naval plane. Palpable contractions occurred at intervals of 1–3 min with a duration of 20–30 s. Vaginal bleeding suggested threatened abortion. Bedside ultrasonography revealed a single live fetus in the uterus. Subsequently, the vaginal bleeding gradually increased, and on the fourth day of admission, a stillborn fetus was delivered. Bedside ultrasonography revealed no obvious tissue residue in the uterine cavity after miscarriage. The patient's vaginal bleeding gradually decreased, and the uterine fundus was located between the umbilicus. On the second day after the miscarriage, the patient had little dark-red vaginal blood without active bleeding. On the seventh day after the miscarriage, the patient had little bloody vaginal secretion with no obvious odor. On the 21st day after the abortion, the patient's uterus essentially recovered, and there was little intermittent light red vaginal discharge with no discernible odor. The vagina had no discernible secretions at the time of hospital discharge.

**Figure 1:**
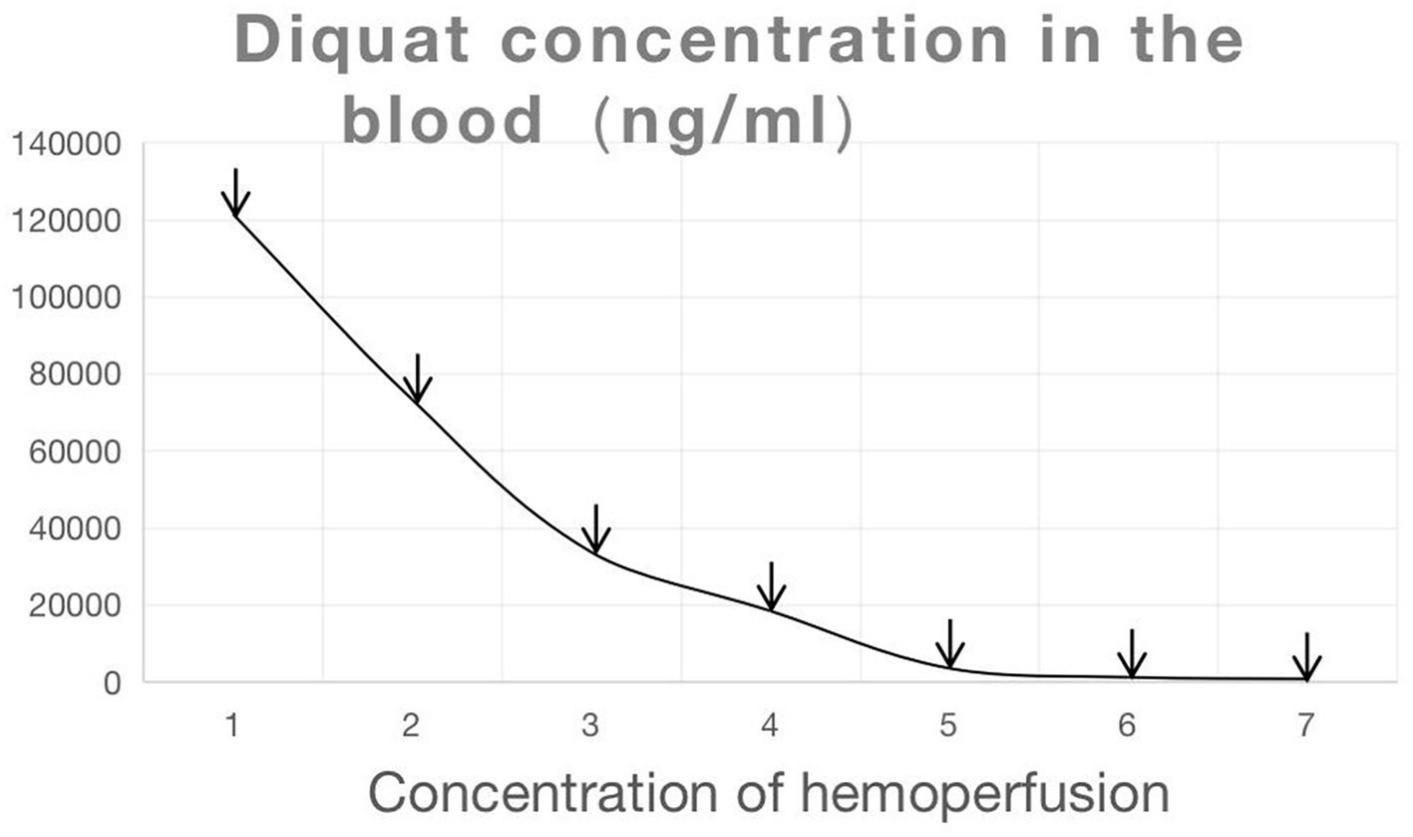
Changes in diquat concentration (The arrows represented that the patient underwent blood purification on that day).

**Figure 2:**
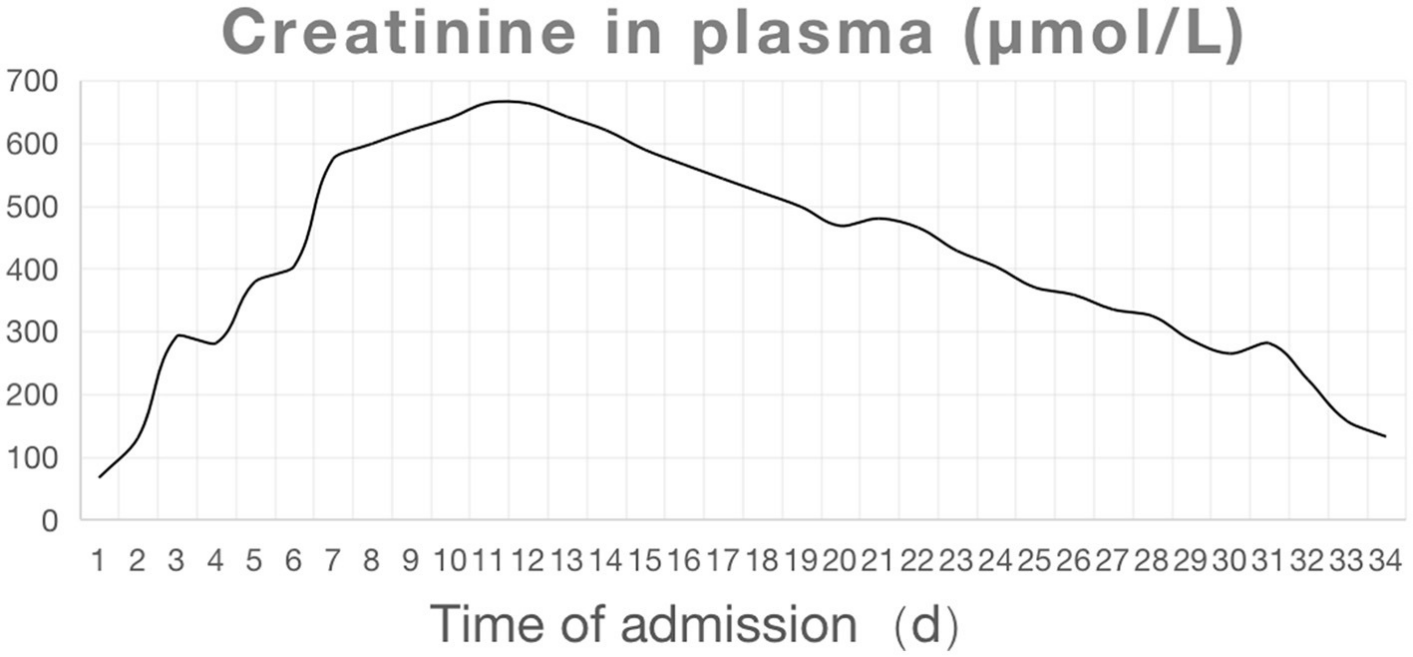
Trends of creatinine levels during the patient's hospitalization.

**Figure 3:**
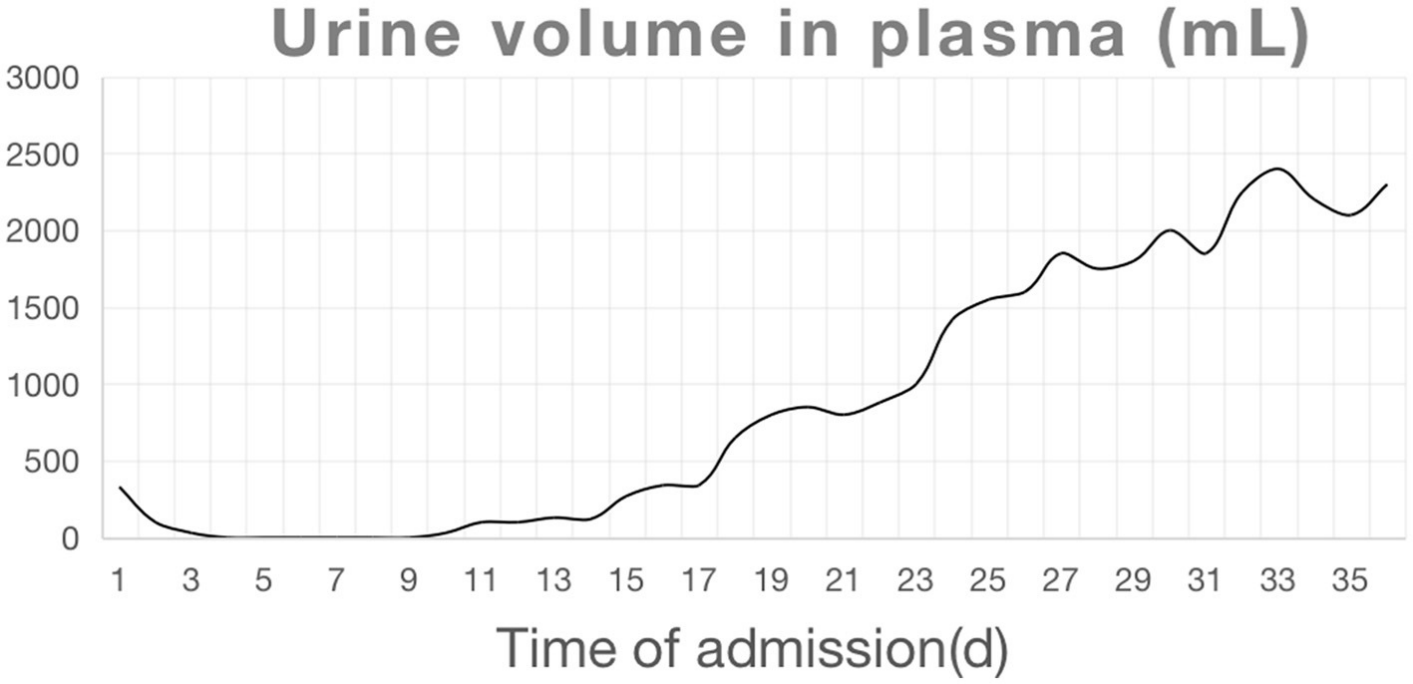
Trends of urine output during the patient's hospitalization.

On admission, the blood concentration of diquat was measured using ultra-high-performance liquid chromatography tandem mass spectrometry (Figure 1 and Table 1). The patient experienced pain and swelling in both lower extremities. Her plasma creatine kinase level was 22,154 U/L (Figure 2), and her 24 h urine output was 330 mL on the first day of admission, which gradually decreased. The patient eventually developed anuria, and her creatinine levels progressively increased. Based on the patient's clinical manifestations and the results mentioned above, she was diagnosed with rhabdomyolysis and acute renal failure. On admission, the patient underwent bedside blood purification. On the 10th day of admission, the patient began to urinate (24 h urine output, 30 mL). On the 13th day, the patient's creatinine levels gradually decreased. The patient was discharged 37 days after admission. At the time of discharge, the patient's creatinine level was 132 μmol/L, and the 24 h urine volume was 2,300 mL (Figures 1, 3).

**Table 1:** Concentration of myoglobin during the patient's hospitalization.

Time of admission (d)	1	2	3	9	10	11	12	17	18
MYO (ng/mL)	240.2	560.4	426.4	>3,000	>3,000	>3,000	2,759	1,492	1,675

**Figure 4:**
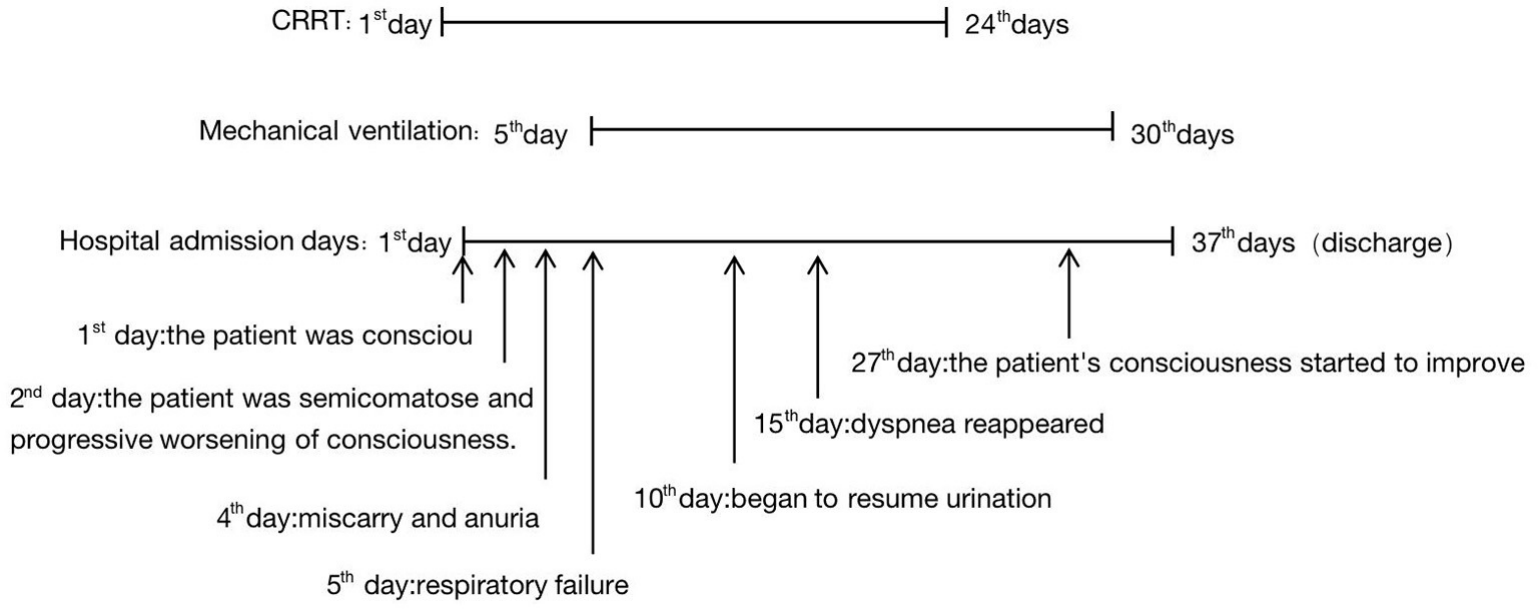
Patient's duration of treatment and significant change during the patients's hospitalization.

The patient was conscious on the day of admission and had a Glasgow Coma Scale (GCS) score of 15 and a Glasgow–Pittsburgh Cerebral Classification (CPC) score of 1. On the second day of admission, the patient was semicomatose, with a GCS score of 7, a CPC score of 4, and progressive worsening of consciousness. On the 5th day of admission, the patient was extubated and placed on mechanical ventilation because of respiratory failure. Dyspnea reappeared on the 15th day of admission, leading to the initiation of mechanical ventilation support following tracheotomy. On the 27th day of admission, the patient's consciousness started to improve. The tracheal tube was closed on the 30th day after admission. The patient was discharged on the 37th day of admission. She was unresponsive, with a GCS score of 12 and a CPC score of 3. The patient was followed up 1 month after discharge, and the patient's consciousness was better than before, with a satisfactory response, the ability to independently finish daily activities, and a CPC score of grade 2 (Figure 4).

## 3 Discussion

Diquat is a non-selective and fast-acting herbicide that is orally absorbed through the gastrointestinal tract. Diquat is excreted mainly through the digestive tract and kidneys. The toxic mechanism is primarily related to the oxidation-reduction process, leading to oxidative stress and cell death, ultimately resulting in multi-organ failure (3).

In severe cases of diquat poisoning, multiple organ damage occurs shortly after ingestion, affecting the liver, kidney, digestive, respiratory, and nervous system. Clinical manifestations and prognosis are related to the dose. In this case, a pregnant woman (15 weeks of gestation) rapidly developed acute renal failure, rhabdomyolysis, toxic encephalopathy, and miscarriage after self-administration of 200 mL of diquat. In a search for documented cases of diquat poisoning (4–9), complications that occurred simultaneously were not documented in previous reports.

In a previous case of diquat poisoning in a pregnant woman (15 weeks of gestation) (7), she miscarried 23 h after ingestion and died 44 h later. The concentration of diquat in the patient's blood 23 h after ingestion was 1.2 lg/mL. The diquat concentrations in the placental blood, umbilical cord blood, and amniotic fluid were 0.8 lg/mL, 0.7 lg/mL, and 0.4 lg/mL, respectively, which showed that diquat could cross the placenta and could be lethal to the fetus. However, information on the toxic effects of diquat on the female reproductive system is limited. Long-term exposure to diquat can impair oocyte function by improving the expression of apoptotic genes and enhancing ROS levels and can also affect fetal development and litter sizes (10). Diquat also causes embryonic malformations in humans (11). In this case, the pregnant woman experienced a miscarriage on the 4th day of admission, which may be attributed to the ability of diquat to cross the placenta and induce oxidative stress and cytotoxicity, ultimately leading to fetal demise. Fortunately, it has been reported that she is the only pregnant woman to have survived the poisoning.

Currently, no specific antidote exists for diquat poisoning. For patients who ingest diquat orally, treatment focuses on decreasing absorption and enhancing elimination. Gastric lavage, medicinal charcoal adsorption, oral mannitol, and other treatments can facilitate diquat excretion. Gastric lavage performed as early as possible in patients with oral intoxication, especially within 1 h, has the best effect on removing the poison, whereas gastric lavage can still be considered within 6 h for those with gastroparesis or considerable intake (2). In this case, the patient presented to a local hospital and underwent gastric lavage 1 h after ingesting diquat. Owing to the significant oral intake, the patient underwent gastric lavage again at our hospital. Blood purification is an effective choice to relieve the poisoning of diquaty. In this case, 13 blood purification treatments were performed, including 6 hemoperfusions and 13 renal replacement therapies. These treatments can maintain the stability of the internal environment and fluid balance during the recovery period of renal function. Animal experiments have indicated that diquats cause renal tubular degeneration and necrosis (12). Diquat is primarily metabolized by the kidneys and can easily cause acute kidney injury. In this case, in addition to acute kidney injury caused by diquat, rhabdomyolysis also exacerbated the condition. Diquat can also damage the central nervous system cells (13). Severe brain damage may be associated with higher mortality rates in patients with diquat poisoning (14). Although the mechanism of toxicity is not fully understood, research indicates that reduction-oxidation (redox) cycling may play a significant role (15, 16). The use of antioxidant drugs, such as glutathione, may alleviate the neurotoxic effects of diquat (17). In this instance, the patient developed disturbances in consciousness on the second day of drug administration. Subsequent head computed tomography revealed brainstem edema. She gradually regained consciousness with further treatment; however, she exhibited neurological sequelae.

The prognosis of patients is significantly correlated with the amount of poison administered, the timing of the first gastric lavage, blood purification, adsorption, and diarrhea. Decontamination of the digestive tract and blood purification should be performed immediately. Monitoring the concentration of toxins in the blood is recommended.

## Data Availability

The original contributions presented in the study are included in the article/supplementary material, further inquiries can be directed to the corresponding author.
